# Open repair of iatrogenic complete aortic occlusion during robotic right nephrectomy: a case report

**DOI:** 10.1093/jscr/rjaf405

**Published:** 2025-06-16

**Authors:** Faisal Almudaiheem, Hussein Alkohlani, Abdulaziz Alshawmar

**Affiliations:** Department of Vascular Surgery, King Abdulaziz Medical City, National Guard Health Affairs, Riyadh 14611, Saudi Arabia; Department of Vascular Surgery, King Abdulaziz Medical City, National Guard Health Affairs, Riyadh 14611, Saudi Arabia; Department of Vascular Surgery, King Abdulaziz Medical City, National Guard Health Affairs, Riyadh 14611, Saudi Arabia

**Keywords:** aorta injury, aorta repair, vascular complication, aortic occlusion

## Abstract

Vascular injury during nephrectomy is a rare but serious complication. We present a unique case of complete occlusion of the infrarenal aorta following a right-sided robotic nephrectomy. We present a 54-year-old male with no known comorbidities who underwent a right robotic radical nephrectomy with retroperitoneal lymph node dissection for a right renal mass. Postoperatively, the patient developed progressive bilateral lower limb weakness and sensory deficits. Clinical examination revealed absent lower limbs pulses bilaterally, with no Doppler signals. Urgent computed tomography (CT) angiography revealed complete occlusion of the infrarenal. Aortic exploration revealed Hemolok clips clamping the aorta. Arterial occlusions may present with delayed signs and require a high index of suspicion for diagnosis. CT angiography is crucial in guiding management. Prosthetic grafting is the preferred method of repair. Here, we highlight the importance of early recognition and intervention. Prompt surgical revascularization can significantly improve prognosis and recovery.

## Introduction

Vascular injury during radical nephrectomy has been mentioned in the literature since its description 50 years ago [1]. Most studies have reported injuries to the visceral arteries, such as the superior mesenteric artery and celiac trunk [2, 3]. With advancements in surgical imaging and innovations in the fields of laparoscopy and robotics, the rate of complications has decreased, resulting in better patient outcomes [4]. Even though it is rare, the close anatomical relation between the left kidney and the visceral branches can lead to these catastrophic complications [5]. Aortic injury has been described in one case report [6]. Herein, we report a case of complete occlusion of the infrarenal aorta that occurred after right-sided robotic nephrectomy.

## Case presentation

**Figure 1:**
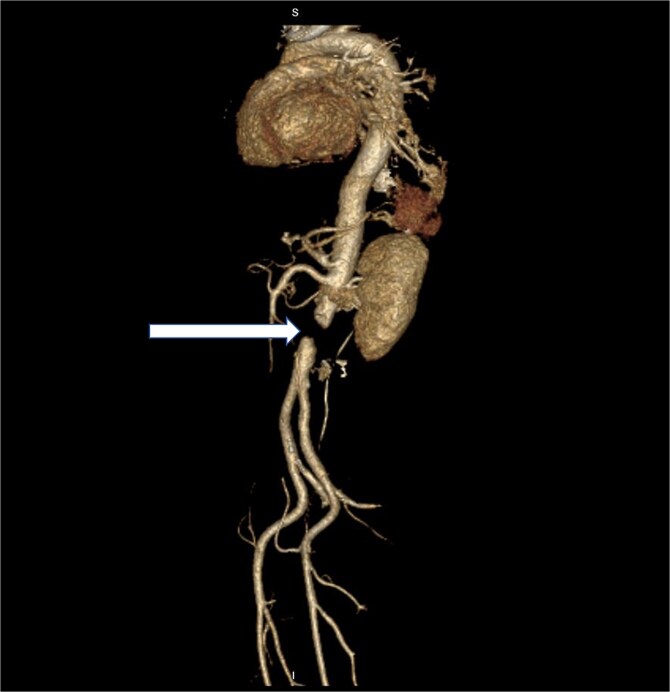
3D reconstructed postoperative CT scan of the abdomen with the arrow showing complete aortic occlusion.

This is a 54-year-old male, not known to have any medical illness, has been investigated by the urology team for a right kidney mass suspicious for renal cell carcinoma, and has undergone an uneventful right robotic radical nephrectomy with retroperitoneal lymph node dissection. The patient was transferred to the surgical intensive care unit for postoperative monitoring. A few hours after the surgery, the patient complained of left lower limb weakness and decreased sensation, followed shortly by similar symptoms in the other limb. Upon examination, the patient was conscious and alert and had stable vital signs, and the lower limb exam revealed absent pulses over bilateral femoral, popliteal, dorsalis pedis, and posterior tibial arteries, with no signals over Doppler. Furthermore, the patient also had motor and sensory deficits. The patient’s postoperative laboratory workups were all unremarkable. An urgent computed tomography scan with contrast was obtained (Fig. 1). A decision for urgent aortic exploration was made, and the patient was shifted to the operating room immediately.

**Figure 2:**
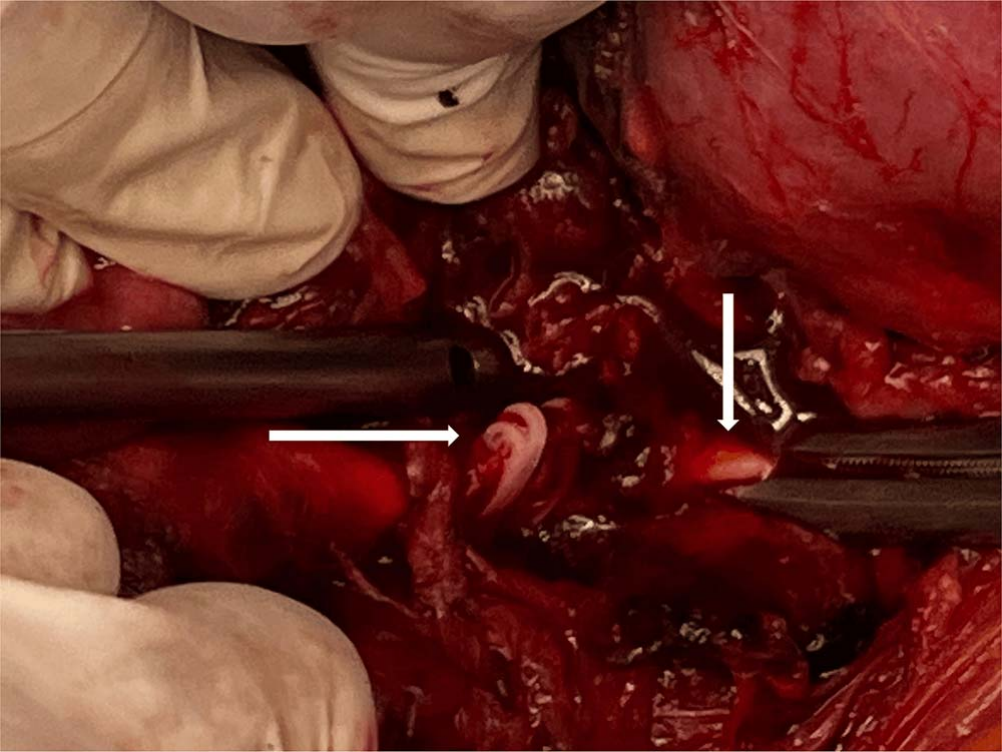
Hemolock clips occluding the aorta.

**Figure 3:**
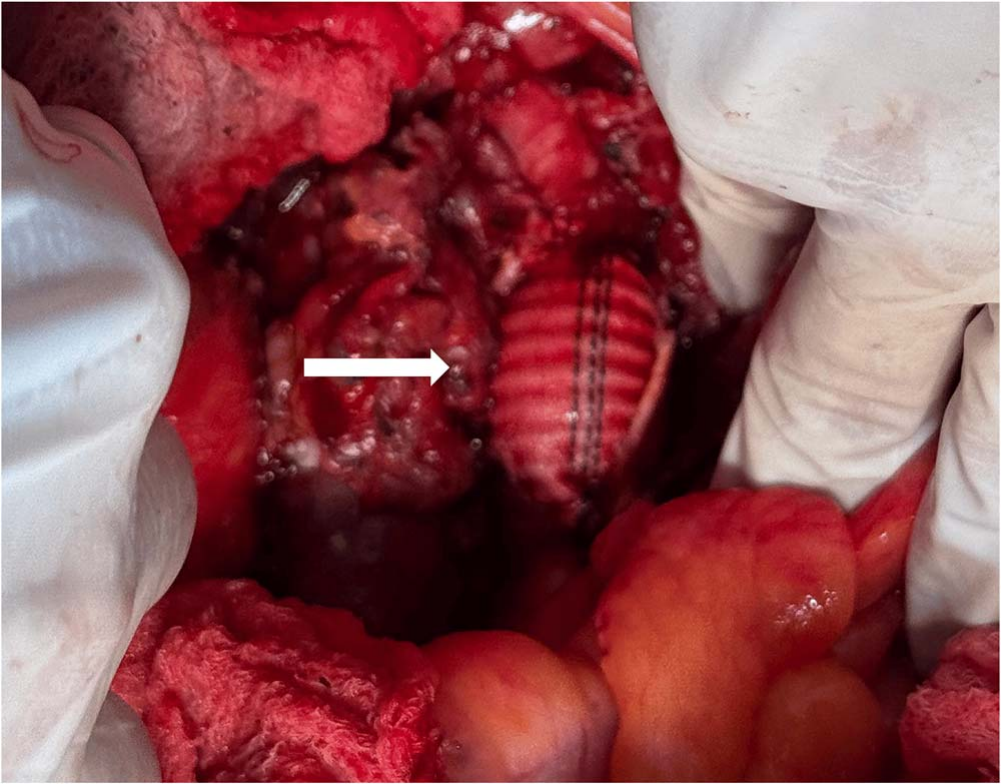
Interposition Dacron graft.

Intraoperatively, an exploratory laparotomy revealed Hemolock surgical clips clamping the infrarenal aorta just above the origin of the inferior mesenteric artery (Fig. 2). Proximal and distal control of the infrarenal aorta was attained. Using bone shears to cut the hemolock clips revealed a complete injury to the aortic wall; a decision was made to ligate the IMA and to perform an interposition graft using a size 16 Dacron graft in an end-to-end fashion (Fig. 3). After completing the anastomosis in the standard fashion and restoring inflow, bilateral pulses in the femoral and lower limb arteries were palpable. Completion of the surgery with the placement of a Jackson-Pratt drain, closing the mesentery, and abdomen was done in the standard manner. Since an expedited restoration of inflow was attained and close postoperative monitoring was implemented, a decision was made not to perform a prophylactic fasciotomy. Postoperative course was uneventful and patient was discharged on postoperative day 6 in a stable condition.

## Discussion

During right radical nephrectomy, an injury to the IVC is more likely, as reported by McAllister *et al.* [7]. However, vascular complications in general can happen during any procedure. Most of the literature concerning impediments from nephrectomy focuses on vascular or hemorrhagic complications. Arterial vascular injury or transection has been reported, mainly to the visceral arteries [2, 3]. Other vascular injuries are usually reported as bleeding or hemorrhage encountered during the surgery or in the postoperative period, requiring intervention or transfusion; it is estimated to occur in 15%–25% of cases [8, 9]. It is easy to observe the bleeding intraoperatively. Clinical signs in cases of complete occlusion might present late, and a delayed diagnosis can increase morbidity and mortality [10]. History and physical exam are vital; duplex ultrasound can be performed at the bedside, and when readily available, CTA is essential to determine the level of injury and plan the management [11]. Aortic injury repair depends on the affected level and extent of the injury. Fortunately, in this patient, only a small area of the infrarenal aorta was affected, but due to extensive wall injury, reconstruction with a prosthetic graft is the preferred method of arterial reconstruction [12], with IMA ligation if necessary, which is usually well tolerated without dire consequences [13]. Prophylactic fasciotomy was not performed, as recent research suggests a more conservative approach to preserve renal function and reduce wound complications [14].

## Conclusions

Vascular injuries during nephrectomy remain a rare but recognized complication. While intraoperative bleeding is often immediately identified and managed, vascular occlusion can present late with grave consequences. A high index of suspicion is needed to diagnose and guide management. Prompt intervention and revascularization can greatly impact outcomes.
